# Association of Apolipoprotein E ɛ4, Educational Level, and Sex With Tau Deposition and Tau-Mediated Metabolic Dysfunction in Older Adults

**DOI:** 10.1001/jamanetworkopen.2019.13909

**Published:** 2019-10-23

**Authors:** Vijay K Ramanan, Anna M. Castillo, David S. Knopman, Jonathan Graff-Radford, Val J. Lowe, Ronald C. Petersen, Clifford R. Jack, Michelle M. Mielke, Prashanthi Vemuri

**Affiliations:** 1 Department of Neurology, Mayo Clinic–Rochester, Rochester, Minnesota; 2 Department of Health Sciences Research, Mayo Clinic–Rochester, Rochester, Minnesota; 3 Department of Radiology, Mayo Clinic–Rochester, Rochester, Minnesota

## Abstract

**Importance:**

While amyloidosis is an early event in the Alzheimer disease (AD) biomarker cascade, a complex interplay among the apolipoprotein E (*APOE*) ɛ4 allele, educational levels, and sex may be associated with an individual’s resilience to dementia.

**Objective:**

To assess whether *APOE* ɛ4, educational levels, and sex are associated with regional tau deposition and tau-mediated metabolic dysfunction in older adults.

**Design, Setting, and Participants:**

Population-based cohort study of individuals aged 65 years and older enrolled between January 1, 2004, and May 1, 2018, in the Mayo Clinic Study of Aging, a prospective longitudinal study of cognitive aging in Olmsted County, Minnesota.

**Main Outcomes and Measures:**

The primary outcomes were cross-sectional tau burden and the fluorodeoxyglucose (FDG) to tau ratio (as a measure of tau-mediated metabolic dysfunction) assessed by positron emission tomography for 43 atlas-defined regions, with specific focus on the entorhinal, inferior temporal, and posterior cingulate cortices.

**Exposures:**

Using linear regression, *APOE* ɛ4 status and years of education were the primary exposure variables, with sex additionally investigated through interaction models.

**Results:**

The sample included 325 individuals (173 [53%] male; mean [SD] age, 76.1 [7.2] years; 291 [90%] cognitively unimpaired). Although *APOE* ɛ4 was nominally associated with higher tau deposition (β = 0.05 [95% CI, 0.02-0.09]; *P* = .001; Cohen *d* = 0.40) and lower FDG to tau ratio (β = −0.05 [95% CI, −0.08 to −0.01]; *P* = .008; Cohen *d* = 0.33) in the entorhinal cortex, these associations were completely attenuated after controlling for global amyloid burden. Education was not associated with regional tau burden or FDG to tau ratio. In the 3 regions of interest, global amyloid burden accounted for the largest proportion of variance in tau deposition among the candidate variables assessed. In the entorhinal cortex, significant interactions were identified between *APOE* ɛ4 and global amyloid burden on tau (β = 0.25; SE = 0.06; *P* < .001) and between sex and tau burden on FDG metabolism (β = 0.10; SE = 0.05; *P* = .049).

**Conclusions and Relevance:**

These results suggest that (1) tau deposition is most significantly associated with amyloidosis; (2) in the presence of abundant amyloidosis, *APOE* ɛ4 may be associated with accelerated entorhinal cortex tau deposition; and (3) women may have lower resilience to tau, manifested by a higher degree of metabolic dysfunction in the entorhinal cortex in response to tau pathology.

## Key Points

**Question:**

Are the apolipoprotein E ɛ4 allele, educational levels, and sex associated with tau deposition and tau-mediated metabolic dysfunction in older adults?

**Findings:**

In a population-based cohort study, regional tau deposition was most significantly associated with global amyloid burden without any main associations of apolipoprotein E ɛ4, education, or sex. Via interaction models, women displayed a higher degree of tau-mediated metabolic dysfunction in the entorhinal cortex compared with men.

**Meaning:**

These findings suggest that in older adults, tau deposition is most significantly associated with amyloidosis, but other factors, including sex, may be associated with differential resilience to tau pathology.

## Teaser

This cohort study investigates the association of the apolipoprotein E (*APOE*) ɛ4 allele, education levels, and sex with tau deposition and tau-mediated metabolic dysfunction in older adults.

## Introduction

The precise mechanistic interactions between the defining proteinopathies of Alzheimer disease (AD), amyloid and tau, are still elusive. According to a widely accepted model of AD pathophysiology, neocortical amyloidosis is hypothesized to occur independently on a background of age-related tauopathy, inducing or facilitating the spread of pathologic tau, which ultimately leads to neurodegeneration resulting in clinical symptoms.^1^ Measures of these pathophysiologic processes in living persons are reflected in the amyloid/tau/neurodegeneration (A/T/N) classification scheme for neuroimaging biomarkers, which provides a research framework for investigation of the potential associations among these biomarkers, as well as the effects of putative AD susceptibility factors.^2,3,4^

It is well understood that the apolipoprotein E (*APOE*) ε4 allele is the strongest known genetic risk factor for sporadic AD. From a biomarker standpoint, *APOE* ε4 has been associated with increased amyloid burden and decreased fluorodeoxyglucose (FDG) metabolism assessed via positron emission tomography (PET).^5,6,7,8,9,10^ However, the association of *APOE* ε4 with tau is less clear. An amyloid-independent effect of *APOE* on tau has been suggested by studies in cultured neurons and animal models,^11,12,13^ but whether this hypothesis holds in vivo in humans is less clear.

Education may protect against the detrimental effects on FDG metabolism associated with *APOE* ε4,^7,14^ and it has been hypothesized that higher education may guard against neurodegeneration and cognitive decline by mitigating tau pathology.^15,16,17^ More broadly, these notions complement the concept of reserve, whereby education and other lifestyle and inherited factors may contribute to differences in brain structure and function that modulate resistance against the development of neurodegenerative pathology and resilience (coping) in the face of pathology.^18,19^

In addition, mounting evidence supports sex-associated differences in risk of AD and its underlying pathophysiology.^20,21,22,23^ Specifically, several reports^24,25,26^ have described sex-specific associations of *APOE* ε4 with tau biomarkers from cerebrospinal fluid. A recent study identified elevated entorhinal cortex tau deposition, measured by PET, among cognitively unimpaired women with elevated amyloid burden compared with men.^27^ Whether these findings extend to the general population of older adults is not known.

In this study, we analyzed cross-sectional PET neuroimaging from a population-based sample of older adults to assess whether *APOE* ε4 and education (as a surrogate measure of resilience) are associated with tau deposition and tau-mediated metabolic dysfunction. We also examined whether sex differences modify these associations.

## Methods

### Selection of Participants

The Mayo Clinic Study of Aging is a population-based prospective study among residents of Olmsted County, Minnesota.^28,29^ Starting in 2004, Olmsted County residents aged 70 to 89 years were identified for recruitment using the Rochester Epidemiology Project medical records linkage system.^30,31^ In 2012, the study was extended to include those aged 50 years and older. Clinical data (through questionnaires and in-person history), neuropsychological assessment, and neuroimaging were assessed at selected visits. Clinical diagnoses were made by an expert consensus panel, incorporating all available information. Data for these analyses were collected between January 1, 2004, and May 1, 2018. All study protocols were approved by the Mayo Clinic and Olmsted Medical Center institutional review boards. Written informed consent was obtained from all participants or their surrogates. Our inclusion criteria included individuals with concurrent tau, FDG, and amyloid PET scans, genotype data for *APOE* allele status, and age 65 years or older based on A/T/N biomarker distributions in cognitively unimpaired individuals.^3^ Using these criteria, we identified 325 elderly individuals for this study. This article was prepared and formatted consistent with the Strengthening the Reporting of Observational Studies in Epidemiology (STROBE) reporting guideline for cohort studies.

### Demographic and Clinical Data

Age, sex, and years of education for each patient were ascertained at a clinical visit. The Short Test of Mental Status (maximum score 38) was used as a summary screen of performance in a variety of cognitive domains. *APOE* allele status (ε2, ε3, ε4) was determined through standard genotyping methods on blood samples.^32^ As a measure of cerebrovascular disease risk, an index score of chronic late-life cardiac, vascular, and metabolic conditions (CMC) was ascertained from health care records as a summation of the presence or absence of hypertension, hyperlipidemia, cardiac arrhythmias, coronary artery disease, congestive heart failure, diabetes, and stroke.^33^

### Neuroimaging Data

The acquisition, processing, and summary measure details for imaging biomarkers assessed from PET scans acquired on the Mayo Clinic Study of Aging participants are described in detail elsewhere.^34^ All analyses used an in-house fully automated image processing pipeline with atlas-defined regions of interest (ROIs) propagated from an MRI template. Amyloid PET imaging was performed with Pittsburgh compound B.^35^ The main amyloid PET measure used for analysis was global amyloid load, computed for each participant by calculating median tracer uptake in the prefrontal, orbitofrontal, parietal, temporal, anterior cingulate, and posterior cingulate/precuneus ROIs, divided by the median uptake in the cerebellar crus gray matter ROI to yield a standardized uptake value ratio (SUVR). Amyloid status was used as a secondary outcome measure, with positivity (vs negativity) defined by SUVR of 1.48 or greater, as previously described.^36^ Tau PET was performed with AV1451, synthesized on site using the precursor compound supplied by Avid Radiopharmaceuticals.^37^ Regional tau burden was computed from median uptake in 43 ROIs divided by the cerebellar crus gray matter ROI. For FDG PET, regional metabolism was computed from median FDG uptake in these same ROIs, normalized by the median uptake in the pons. While 43 ROIs were evaluated, we focused specifically on 3 ROIs known to exhibit early and prominent tau pathology in AD, the entorhinal, inferior temporal, and posterior cingulate cortices.^37,38^

### Statistical Analysis

A combination of software packages was used for analyses, including SPSS Statistics version 22.0 (IBM Corp), RStudio: Integrated Development for R (RStudio Inc), and SAS version 9.4 (SAS Institute Inc). Two-sided significance was set at α = .05 (type I error rate). Standard summary measures were used to describe demographic and clinical characteristics for the sample, stratified by *APOE* ε4 allele status, with group comparisons obtained through *t* tests for continuous variables and χ^2^ tests for categorical variables.

#### Main Analyses

The primary outcome measures for this study were regional tau SUVR and, as a measure of tau-mediated metabolic dysfunction, the ratio of regional FDG to regional tau. These measures were assessed for linearity and outliers using scatterplots and for normality using histograms. Regional tau SUVR values were transformed by natural log to ensure a more normal distribution, while regional FDG to tau ratio measures were analyzed without transformation based on histograms revealing a more normal distribution. Initial models assessed for associations of *APOE* ɛ4 status (0 copies = negative, 1 or 2 copies = positive) and education (expressed as total years), with these outcomes using linear regression. Age, sex, and CMC were included as covariates in all primary models, with global amyloid burden (transformed by natural log to ensure a more normal distribution) as an additional covariate for secondary models. To account for multiple comparisons, the threshold for significance was defined as *P* < .001 based on the Bonferroni correction for 43 regions assessed (.05 / 43 = .0012).

#### Secondary Analyses on ROIs of Focus

Additional analyses were performed for the 3 specific ROIs of focus. For* APOE* ɛ2, ɛ3, and ɛ4 associations, an analysis of covariance model was used to compare *APOE* allele types (ɛ3/ɛ3 vs ɛ2/ɛ2, ɛ2/ɛ3, ɛ2/ɛ4, ɛ3/ɛ4, ɛ4/ɛ4) regarding tau and the FDG to tau ratio in these regions using the least-squares difference for multiple comparisons and including age, sex, and CMC as covariates.

In the full sample, linear regression using stepwise forward entry was used to examine the independent variance explained by significantly associated candidate predictor variables on regional tau burden (age, sex, CMC, *APOE* ɛ4, education, and global amyloid burden) and FDG metabolism (age, sex, CMC, *APOE* ɛ4, education, global amyloid burden, and regional tau burden). For these models, regional FDG metabolism was analyzed without transformation owing to histograms revealing a normal distribution.

To assess whether amyloid status (positive vs negative) affected the association tests for *APOE* ɛ4 described in the primary analyses, the main regression models for regional tau and the FDG to tau ratio were repeated after stratifying the sample by amyloid status.

We examined for several interactions using the following regression models: (1) regional tau = age + sex + CMC + global amyloid + *APOE* ɛ4 + global amyloid × *APOE* ɛ4; (2) regional tau = age + sex + CMC + global amyloid + education + global amyloid × education; (3) regional tau = age + sex + CMC + global amyloid + global amyloid × sex; (4) regional FDG = age + sex + CMC + regional tau + regional tau × sex; (5) regional FDG = age + sex + CMC + *APOE* ɛ4 + sex × *APOE* ɛ4; and (6) regional FDG = age + sex + CMC + education + education × sex.

## Results

Characteristics of the 325 participants are summarized in Table 1. Mean (SD) age was 76.1 (7.2) years; 173 participants (53%) were men; and 291 (90%) were cognitively unimpaired. As expected, *APOE* ɛ4–positive individuals displayed higher global amyloid burden compared with *APOE* ɛ4–negative counterparts (SUVR, 1.84 [95% CI, 1.74-1.94] vs 1.57 [95% CI, 1.52-1.61]; *P* < .001). After stratification by *APOE* ɛ4 status, study participants did not differ with regard to age, sex, years of education, CMC, or cognitive status.

**Table 1.** Summary Characteristics of the Sample Stratified by *APOE* ɛ4 Allele Status

Characteristic	No. (%)		*P* Value^a^
	*APOE* ɛ4 Negative (n = 232)	*APOE* ɛ4 Positive (n = 93)	
Age, mean (SD), y	76.1 (7.5)	76.2 (6.5)	.90
Men	126 (54.3)	47 (50.5)	.54
Education, mean (SD), y	14.6 (2.5)	14.9 (2.4)	.32
CMC score, mean (SD)^b^	2.2 (1.5)	2.3 (1.4)	.46
Cognitively unimpaired	208 (89.7)	83 (89.2)	.91
Mild cognitive impairment	20 (8.6)	7 (7.5)	.75
Dementia	3 (1.3)	3 (3.2)	.24
Short Test of Mental Status score, mean (SD)^c^	35.1 (2.9)	35.1 (2.7)	.96
Global amyloid PET SUVR, mean (SD)	1.57 (0.38)	1.84 (0.49)	<.001

Abbreviation: *APOE*, apolipoprotein E; CMC, cardiovascular and metabolic conditions; PET SUVR, positron emission tomography standardized uptake value ratio.

^a^ Via *t* test for continuous variables and χ^2^ test for categorical variables.

^b^ Index score of cerebrovascular disease risk.

^c^ Total score (maximum 38); data missing for 1 patient who was *APOE* ɛ4 negative.

### Main Analyses

**Figure 1.**
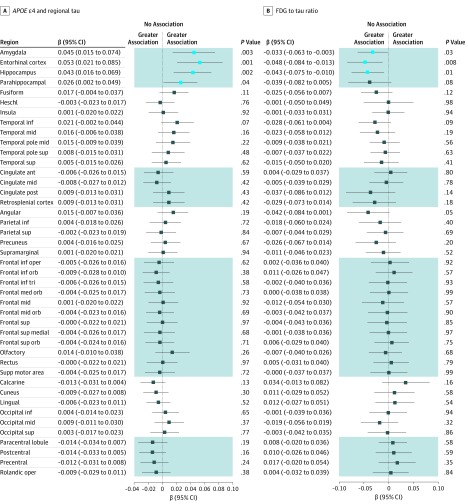
Association of *APOE* ɛ4 With Regional Tau and Tau-Mediated Metabolic Dysfunction A, Associations for *APOE* ɛ4 with regional tau. B, Tau-mediated metabolic dysfunction modeled as the fluorodeoxyglucose (FDG) to tau ratio. Age, sex, and an index score of cerebrovascular disease risk were included as covariates. Light blue boxes indicate uncorrected *P* < .05; dark blue boxes, uncorrected *P* ≥ .05; shading, contiguous regions; ant, anterior; inf, inferior; mid, middle; oper, operculum; orb, orbital; sup, superior; and tri, triangularis.

**Figure 2.**
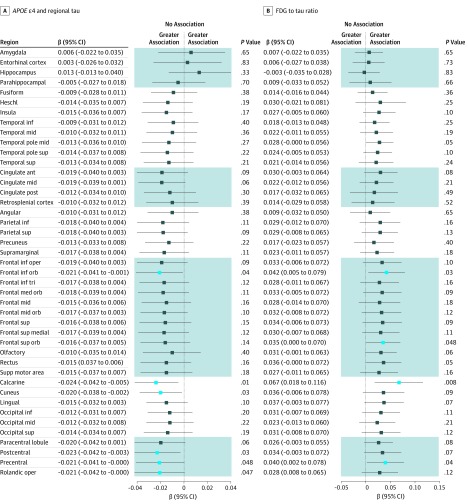
Associations of *APOE* ɛ4 With Regional Tau and Tau-Mediated Metabolic Dysfunction Attenuate After Controlling for Amyloidosis A, Associations for *APOE* ɛ4 with regional tau. B, tau-mediated metabolic dysfunction modeled as the fluorodeoxyglucose (FDG) to tau ratio. Associations include global amyloid burden as a covariate in addition to age, sex, and an index score of cerebrovascular disease risk, which were included in Figure 1. Light blue boxes indicate uncorrected *P* < .05; dark blue boxes, uncorrected *P* ≥ .05; shading, contiguous regions; ant, anterior; inf, inferior; mid, middle; oper, operculum; orb, orbital; sup, superior; and tri, triangularis.

In the primary main effect analyses (Figure 1), the presence of the *APOE* ɛ4 allele was nominally associated with higher tau burden in the entorhinal cortex (β = 0.05 [95% CI, 0.02-0.09]; *P* = .001; Cohen *d* = 0.40), hippocampus (β = 0.04; [95% CI, 0.02-0.07]; *P* = .002; Cohen *d* = 0.38), amygdala (β = 0.05 [95% CI, 0.02-0.07]; *P* = .003; Cohen *d* = 0.37), and parahippocampal gyrus (β = 0.03 [95% CI, 0.002-0.05]; *P* = .04; Cohen *d* = 0.26). The presence of the *APOE* ɛ4 allele was also nominally associated with a lower FDG to tau ratio (β = −0.05 [95% CI, −0.08 to −0.01]; *P* = .008; Cohen *d* = 0.33) in the entorhinal cortex, suggesting more metabolic dysfunction relative to tau burden in that region for participants with the ɛ4 allele vs those without. However, all of these findings were completely attenuated after controlling for global amyloid burden (Figure 2). Education was not associated with tau burden or the FDG to tau ratio in any region.

### Secondary Analyses on ROIs of Focus

Additional investigations focused on 3 regions vulnerable to early and prominent tau deposition in AD, the entorhinal, inferior temporal, and posterior cingulate cortices.

**Figure 3.**
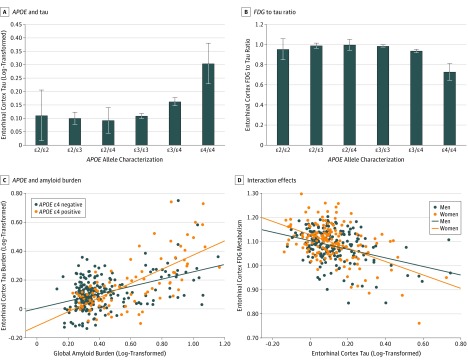
Secondary Analyses of *APOE* Allele Associations and Interaction Models in the Entorhinal Cortex A and B, Compared with individuals who had *APOE* ɛ3/ɛ3, those with *APOE* ɛ3/ɛ4 and ɛ4/ɛ4 displayed higher tau deposition (A) and a lower fluorodeoxyglucose (FDG) to tau ratio (B) via an analysis of covariance model. Error bars indicate standard error of the mean. C, Via interaction studies, in the setting of high global amyloid burden, *APOE* ɛ4–positive individuals (orange) displayed higher entorhinal cortex tau deposition than their *APOE* ɛ4–negative counterparts (blue). D, Via interaction studies, in the setting of high entorhinal cortex tau deposition, women (orange) displayed greater tau-mediated metabolic dysfunction than men (blue).

A breakdown of the data for tau and the FDG to tau ratio by *APOE* allele characterization (ɛ3/ɛ3 vs ɛ2/ɛ2, ɛ2/ɛ3, ɛ2/ɛ4, ɛ3/ɛ4, and ɛ4/ɛ4) was analyzed, with representative results from the entorhinal cortex displayed (Figure 3A and B). In that region, compared with individuals with *APOE* ɛ3/ɛ3 or ɛ3/ɛ4, those homozygous for the *APOE* ɛ4 allele did not have significantly higher tau deposition (difference in estimated marginal means = 0.14 [95% CI, −0.01 to 0.30]; *P* = .07) but did have a significantly lower FDG to tau ratio (difference in estimated marginal means = −0.21 [95% CI, −0.38 to −0.04]; *P* = .02), although these results should be interpreted with caution given the relatively small number of individuals with *APOE* ɛ4/ɛ4 in the sample (n = 3). Presence of the *APOE* ɛ2 allele under dominant (presence vs absence) or additive (0 vs 1 vs 2 copies) conditions was not associated with tau or the FDG to tau ratio in any of the 3 ROIs.

Based on stepwise regressions, among the candidate variables assessed, global amyloid burden accounted for the largest proportion of the variance in tau burden in each of these regions (Table 2). The largest proportion of variance in regional tau burden explained by global amyloid burden was in the entorhinal cortex (change in adjusted *R*^2^ = 0.32; β = 0.34 [95% CI, 0.28-0.39]; *P* < .001). Modest additional contributions to the variance in tau were identified for CMC on entorhinal cortex tau (change in adjusted *R*^2^ = 0.01; β = 0.01 [95% CI, 0.001-0.018]; *P* = .03) and age on inferior temporal cortex tau (change in adjusted *R*^2^ = 0.01; β = 0.001 [95% CI, 0-0.003]; *P* = .049). Entorhinal cortex tau burden accounted for the largest proportion of variance in FDG metabolism in that region (change in adjusted *R*^2^ = 0.18; β = −0.15 [95% CI, −0.21 to −0.09]; *P* < .001), with additional accounting of variance by age (change in adjusted *R*^2^ = 0.06; β = −0.002 [95% CI, −0.003 to −0.002]; *P* < .001) and global amyloid burden (change in adjusted *R*^2^ = 0.02; β = −0.05 [95% CI, −0.09 to −0.01]; *P* = .01). In contrast, in the inferior temporal and posterior cingulate cortices, tau burden did not account for any of the variance in FDG metabolism. Instead, FDG metabolism in those regions was explained by global amyloid burden, age, sex, and CMC, as well as a modest contribution of education for inferior temporal cortex FDG metabolism (Table 2).

**Table 2.** Factors Accounting for the Variance in Tau Burden and FDG Metabolism in Key Alzheimer Disease Regions via Stepwise Regressions

Factor	β (95% CI)	Unique Adjusted *R*^2^	*P* Value
**Tau Models**			
Entorhinal cortex tau			
Global amyloid	0.34 (0.28 to 0.39)	0.32	<.001
CMC score^a^	0.01 (0.001 to 0.02)	0.01	.03
Inferior temporal cortex tau			
Global amyloid	0.20 (0.15 to 0.24)	0.24	<.001
Age	0.001 (0.000 to 0.003)	0.01	.049
Posterior cingulate cortex tau			
Global amyloid	0.14 (0.10 to 0.18)	0.11	<.001
**FDG Models**			
Entorhinal cortex FDG			
Entorhinal cortex tau	−0.15 (−0.21 to −0.09)	0.18	<.001
Age	−0.002 (−0.003 to −0.001)	0.06	<.001
Global amyloid	−0.05 (−0.09 to −0.01)	0.02	.01
Inferior temporal cortex FDG			
Global amyloid	−0.13 (−0.18 to −0.09)	0.13	<.001
Age	−0.002 (−0.004 to −0.001)	0.04	.005
Sex (male)	−0.03 (−0.05 to −0.01)	0.03	.002
CMC score^a^	−0.007 (−0.01 to 0.00)	0.01	.04
Education	0.004 (0.00 to 0.008)	0.01	.04
Posterior cingulate cortex FDG			
Global amyloid	−0.21 (−0.29 to −0.13)	0.12	<.001
Sex (male)	−0.08 (−0.12 to −0.05)	0.08	<.001
CMC score^a^	−0.02 (−0.04 to −0.01)	0.06	<.001
Age	−0.004 (−0.006 to −0.001)	0.02	.004

Abbreviations: CMC, cardiovascular and metabolic conditions; FDG, fluorodeoxyglucose.

^a^ Index score of cerebrovascular disease risk.

Given the strong association of global amyloid burden with regional tau, we assessed whether *APOE* ɛ4 may have differential associations to tau burden and the FDG to tau ratio in amyloid-positive vs amyloid-negative individuals. After stratifying the sample by amyloid status, the presence of the *APOE* ɛ4 allele was nominally associated with higher entorhinal cortex tau deposition (β = 0.059 [95% CI, 0.005-0.113]; *P* = .03) in amyloid-positive individuals but not in amyloid-negative individuals. *APOE* ɛ4 was not associated with tau burden or the FDG to tau ratio in the other regions when stratifying by amyloid status.

We also examined interactions between several pairs of variables for tau burden and FDG metabolism in the 3 ROIs. There was an interaction between the presence of the *APOE* ɛ4 allele and global amyloid burden for entorhinal cortex tau (β = 0.25; SE = 0.06; adjusted *R*^2^ = 0.36 for model; *P* < .001 for amyloid × *APOE* interaction term) such that presence of the *APOE* ɛ4 allele was associated with higher entorhinal cortex tau among individuals with higher global amyloid burden (Figure 3C). There was no significant amyloid × *APOE* interaction on inferior temporal or posterior cingulate cortex tau deposition and no amyloid × sex or amyloid × education interaction on any of the 3 regional tau measures. In the entorhinal cortex, there was an interaction between sex and tau burden on FDG metabolism (β = 0.10; SE = 0.05; adjusted *R*^2^ = 0.24 for model; *P* = .049 for tau × sex interaction term) such that, among individuals with greater tau burden, women displayed lower FDG metabolism compared with men (Figure 3D). There was no interaction between tau and sex for inferior temporal or posterior cingulate cortex FDG and no interactions between sex and *APOE* or education for any of the 3 regional FDG measures.

## Discussion

This population-based cohort study of older adults found that regional tau deposition was associated with global amyloidosis. In addition, in the presence of abundant amyloidosis, *APOE* ɛ4 may have accelerated entorhinal cortex tau deposition. Furthermore, women may have had lower resilience to tau, manifested by a higher degree of metabolic dysfunction in the entorhinal cortex in response to tau pathology.

Although there has been long-standing interest in whether a mechanistic relationship exists between *APOE* and parenchymal tau that is independent of amyloid, evidence for such a relationship has generally been indirect.^39^ Recent studies^12,13^ in model systems of primary tauopathy demonstrated that introduction of *APOE* ɛ4 led to worsened tau pathology and neurodegeneration. Examining postmortem human brains with primary tauopathies, one study^13^ identified an association of *APOE* ɛ4 with greater neurodegeneration after controlling for a variety of pathologies, while a separate report^40^ described an association of *APOE* ɛ2 with increased tau pathology. A genome-wide association study^41^ also suggested an association of the *APOE* locus with cerebrospinal fluid levels of phosphorylated tau that remained significant after controlling for cerebrospinal fluid amyloid levels. One prior study^42^ of 35 individuals identified an association between *APOE* ɛ4 and medial temporal and parietal tau burden assessed voxelwise by PET, though this sample predominantly consisted of individuals with clinically atypical AD.

The findings from our study of a large, population-based sample, which tested parenchymal tau deposition assessed on a regional basis via PET, add valuable context to this debate. It is well known that the *APOE* ɛ4 allele is associated with amyloid accumulation, a process that emerges over decades and precedes overt clinical symptoms in AD.^43^ Current evidence suggests that *APOE* ɛ4 is not associated with suspected nonamyloid pathophysiology and primary age-related tauopathy,^44,45^ supporting that the *APOE* associations in this study are likely along the AD cascade. Overall, our results support a model in which amyloid is significantly associated with tau deposition, but with *APOE* ɛ4 potentially being associated with accelerated tau pathology in key AD regions in the presence of substantial amyloidosis. The latter supposition is supported by our data showing that for a given burden of high amyloid, *APOE* ɛ4 carriers displayed higher entorhinal cortex tau than *APOE* ɛ4 noncarriers (Figure 3C). These findings are consistent with postmortem human neuropathology data describing an association of *APOE* ɛ4 with tau tangle pathology only in the presence of amyloid.^46^ Our data also implicitly mirror presented voxelwise neuroimaging data from a smaller cohort detailing an association of *APOE* ɛ4 with medial temporal lobe tau in patients with AD, but not in cognitively unimpaired individuals for whom amyloid was the factor most significantly associated with tau pathology.^47^ Although speculative, neuroinflammation, including the facilitation by *APOE* of microglial and astrocytic responses to amyloid, could be a mechanistic factor in this complex association.^13,48,49^

We found that in comparison with men, women displayed a greater susceptibility to neurodegeneration (proxied by lower FDG metabolism) in the setting of higher tau deposition in the entorhinal cortex. No similar interaction was identified in the inferior temporal or posterior cingulate cortices, suggesting the possibility of a region-specific association. While there is no strong evidence that older women have higher amyloid burden than older men, based on epidemiological studies of those with mild cognitive impairment, women exhibit faster cognitive decline compared with men,^22,50,51^ suggesting that any relationships of sex with AD-type pathophysiology are likely downstream to amyloidosis. Our findings support this hypothesis by providing evidence that women may have lower resilience to tau compared with men, manifested by a greater degree of metabolic dysfunction at a given level of tau deposition. We anticipate that a better understanding of the roles of sex differences in the mechanisms underlying AD will play a key role in the development of treatments to combat its clinical heterogeneity.^52,53^

We found no associations of education with regional tau or with the relationship of FDG metabolism to tau. Education, a surrogate of resilience, has been shown to delay the onset of cognitive impairment by improving an individual’s ability to cope with AD pathologies. Because a fraction of amyloid-positive individuals remain in asymptomatic/preclinical stages of AD, one line of reasoning is that higher education may mitigate the presence of significant amyloidosis through effects on tau.^15,54^ A recent study of 38 individuals with AD identified an association of educational level with tau burden in regions where tau and FDG hypometabolism overlapped, implying that in individuals with higher education, more tau pathology may be required to yield metabolic dysfunction.^55^ Our results did not identify evidence in support of this specific hypothesis and suggest that mechanisms that explain resilience are not associated with reduction of tau deposition. Indeed, the modest accounting of variance by education on FDG metabolism in the inferior temporal cortex without a concomitant association of education with the FDG to tau ratio in this region further supports the presumption that any association of education with resilience (measured metabolically or otherwise) is likely not tau dependent, and may instead be associated with other factors such as healthier lifestyles and better vascular health.^56^ The difference in conclusions from our results compared with the study by Hoenig and colleagues^55^ may have to do with differences in sample composition (population-based vs individuals with AD), sample size, and accounting for the effects of amyloidosis and cerebrovascular disease risk.

The association of vascular risk with FDG-PET has been widely shown and is consistent with our findings.^57^ There is increasing evidence that vascular risk has weak to modest associations with tau deposition. In separate models, we did observe a modest accounting of variance by cerebrovascular disease risk on entorhinal cortex tau burden after accounting for the association of global amyloid burden, consistent with prior reports^33,58^ that cerebrovascular disease and amyloid pathology can concurrently influence tau burden.

### Limitations

Our study has several limitations. Among current tau PET tracers, the efficacy in assessing non-AD tau, including primary age-related tauopathy, is still unclear and could lead to underestimation of overall tau pathology.^59^ Future studies using a longitudinal design could help to clarify the extent to which medial temporal tau deposits due to distinct (AD and non-AD) etiologies may have differential associations with the variables analyzed in this study. In addition, the development of PET tracers for other proteinopathies of aging and neurodegenerative disease, such as transactive response DNA binding protein 43,^60^ would facilitate noninvasive accounting for the effects of comorbid pathologies. We analyzed education as a surrogate of resilience owing to its extensive use in prior work, but its presumed influence early in life could make it insensitive as a sole measure of reserve and resilience.^61^ Although the association of global amyloid burden with regional tau deposition supports that our sample had sufficiently mature pathology to assess for other associations, the fact that our sample consisted predominantly of cognitively unimpaired older individuals may have limited the dynamic range for discovering associations of predictor variables with outcomes in regions other than the entorhinal cortex. In addition, our analyses of FDG PET cannot rule out the possibility of subtle associations with neurodegeneration captured on MRI by regional volumetric or voxelwise approaches. However, FDG-based resilience studies have been more consistent than MRI-based resilience studies (which may not provide an adequate marker of synaptic health), and therefore this study focused on FDG-based neuronal dysfunction.

## Conclusions

This population-based cohort study of older adults found that regional tau deposition was associated with global amyloidosis. In addition, in the presence of abundant amyloidosis, *APOE* ɛ4 may be associated with accelerated entorhinal cortex tau deposition. Women may have lower resilience to tau, manifested by a higher degree of metabolic dysfunction in the entorhinal cortex in response to tau pathology.

Our findings support a model whereby global amyloid burden is significantly associated with regional tau deposition in older adults. However, *APOE* allele status and sex may be associated with differences in AD-type pathophysiology that are downstream of amyloid and more proximal to clinical symptoms. More broadly, this work highlights the value of the A/T/N framework in hypothesis testing surrounding underlying biological mechanisms and argues for further investigation of the potential intervening steps between early amyloidosis and later features of AD pathophysiology, particularly in the context of the failures thus far of AD clinical trials based on amyloid-lowering strategies.^62^
